# Consent to testing for brain death

**DOI:** 10.1136/jme-2023-109425

**Published:** 2023-10-25

**Authors:** Barry Lyons, Mary Donnelly

**Affiliations:** 1 Trinity College Dublin, Dublin, Ireland; 2 University College Cork, Cork, Ireland

**Keywords:** death, ethics- medical, human rights, informed consent

## Abstract

Canada has recently published a new Clinical Practice Guideline on the diagnosis and management of brain death. It states that consent is not necessary to carry out the interventions required to make the diagnosis. A supporting article not only sets out the arguments for this but also contends that ‘UK laws similarly carve out an exception, excusing clinicians from a prima facie duty to get consent’. This is supplemented by the claim that recent court decisions in the UK similarly confirm that consent is not required, referencing two judgements in *Battersbee*. We disagree with the authors’ interpretation of the law on consent in the UK and argue that there is nothing in *Battersbee* to support the conclusion that consent to testing is not necessary. Where there is a disagreement about testing for brain death in the UK, court authorisation is required.

## Introduction

In May 2023, a new Clinical Practice Guideline on the ‘definition of death and criteria for its determination’ was published in Canada.^1^ Among other elements, it sets out a biomedical definition of brain death (BD) ‘based on permanent cessation of brain function that applies to all persons, as well as recommendations for … death determination by neurologic criteria (DNC)’.^1^ An accompanying editorial described it as ‘a tour de force … a comprehensive, rigorous, and evidence-based approach to updating the Canadian guidelines, which can serve as a model for many other countries to follow’.^2^ The guideline is influenced by the World Brain Death Project (WBDP), which was established to harmonise global practice in the diagnosis and management of BD. The WBDP noted that

Difficulties in conducting randomized clinical trials and large-scale studies on BD/DNC have resulted in a lack of robust data from which to develop evidence-based recommendations. Challenges to the validity of BD/DNC continue to promote controversy.^3^

Notwithstanding the acknowledged paucity of evidence, the WBDP sets down over one hundred recommendations and suggestions, including three relating to consent, positing that consent is not, nor should not be, required for testing for BD, or for the removal of somatic support (ventilator, infusions, etc) in the event that the test for BD is positive. The Canadian Guideline aligns with the WBDP position on consent, with the reasoning of the guideline Consent Working Party published in an accompanying article in the Canadian Journal of Anaesthesia (hereafter ‘CJA article’).^4^

The purpose of our paper is to critique this position on consent to testing for BD, and the justifications provided.^2^ We are concerned especially with a statement in the CJA article that ‘UK laws similarly carve out an exception, excusing clinicians from a prima facie duty to get consent for DNC’ testing. This question is especially important given that the Academy of Medical Royal Colleges (AoMRC) is currently in the process of revising the Code of Practice for the Diagnosis and Confirmation of Death.^5^ Thus, our primary interest lies in examining whether the arguments provided are applicable to the UK,^3^ and our own jurisdiction, Ireland.

## Diagnosing BD

BD is exclusively diagnosed in an intensive care unit setting due to the need for mechanical ventilation. As indicated earlier, there is no universal definition of BD and no globally agreed criteria for its diagnosis. The whole brain formulation (‘irreversible cessation of all functions of the entire brain, including the brain stem’)^6^ applies in North America, most European countries, and Australia and New Zealand. The brainstem version (‘the irreversible loss of the capacity for consciousness, combined with irreversible loss of the capacity to breathe’) applies in the UK and Ireland, with the relevant guidance detailed in the AoMRC Code and the *Diagnosis of Brain Death in Adults Guidelines*, respectively.^7^ Despite their apparent differences, both whole brain and brainstem death formulations effectively depend on similar clinical testing following a judgement that the patient will never recover consciousness after a severe brain injury. Testing includes first, a relatively straightforward assessment of the functioning of the brainstem, and second, the apnoea test, where the patient is disconnected from the ventilator, passively supplied with oxygen, and observed to ascertain whether they make any independent efforts at breathing. In accordance with the Code, where there has been no response to the neurological assessment, a positive apnoea test (no spontaneous respiration) confirms the diagnosis of brainstem death. This is not a universal position—in many European jurisdictions neurophysiological or radiological investigations are required in addition to clinical testing for the diagnosis of BD to be made. There is no statutory definition of death in either the UK or Ireland, and the issue of consent to testing is not mentioned in their respective guidance.

In contrast, the Canadian Guideline contends that ‘consent for DNC testing should neither be required nor requested’, holding that the ‘overwhelming weight of legal and ethical authority holds that clinicians are not required to obtain family consent before performing DNC testing’.^1^ One reason why the guideline addresses the issue of consent is the perceived emergence of resistance to BD testing from the families of afflicted individuals. There has been an upsurge in disagreement or conflict in the context of a diagnosis of DNC,^8^ although the overall numbers remain very small. More specifically, there have been several situations where families or surrogate decision-makers have withheld consent to DNC testing, most publicly in the cases of Archie Battersbee in the UK,^9^ and Taquisha McKitty in Canada.^10^

## Reasons set out against a consent requirement for BD testing

The CJA article sets out several overlapping arguments, both factual and normative, to underpin their contention that consent to BD testing is not required. Those of relevance to our discussion of the position in the UK/Ireland include:

1. There is a near consensus among international professional guidelines and statutes, and court decisions in the USA, Canada and the UK, on this matter. Moreover, prevailing medical practice does not require consent to test for BD. Accordingly, those that propose that consent is required bear the ‘heavy burden’ of responsibility to prove that consent is necessary.
2. Akin to this, the CJA article cites a number of commentators who propose that ‘the prima facie duty to obtain consent is never triggered in the first place’ because BD testing is not treatment^11^; that it is not healthcare per se, but rather a form of assessment or evaluation^12^; that it is simply a means to determine whether healthcare is appropriate,^13^ comparable to assessing a patient’s decision-making capacity.^14^
3. BD represents a situation of ‘unique and special importance’, whereby there is a ‘fundamental need’ to answer the pivotal question as to whether the person is alive or dead according to the accepted standard. The necessity to address this fundamental need outweighs any obligation in respect of consent.
4. In a situation of cardiac arrest, clinicians do not ask for consent to assess whether a patient’s heart has stopped beating or they have stopped breathing. There should be symmetry between how we think about evaluating patients when their heart stops beating and when their brain stops functioning.
5. If doctors cannot assess whether a patient is dead or not then this generates several problems, including those of distributive justice, risks to the ‘integrity of the medical profession’, and moral injury concerns.

## Analysis of CJA article’s reasoning

### Presumption that consent is not required

The suggestion that a prima facie case needs to be made in order to justify that consent is necessary for a medical procedure to take place seems odd from a UK and Irish perspective, representing an inversion of standard processes. It seems trite to declare that the rights to self-determination and bodily integrity in both jurisdictions are legally protected against unconsented medical intervention by common law,^15 16^ the European Convention on Human Rights (Article 8),^17^ and (in Ireland) by the Constitution. This right was described by Lady Justice Hale as ‘the most important of civil rights’.^18^

While the level of touching required to infringe on bodily integrity is uncertain in English law,^19^ it seems clear that what takes place during BD testing would cross any reasonable legal threshold—the deliberate infliction of a painful stimulus, oral intrusion and instrumentation of the pharynx and larynx to provoke gag and cough reflexes, stimulation of the cornea of the eye, injection of iced fluid into the ear canal. The apnoea test, according to the expert witness in *Re Archie Battersbee*, presents a ‘significant physiological challenge’ (at [44]).^9^

There are of course caveats to the standard rules around consent in particular situations such as emergencies, and in cases involving children or adults who lack capacity. Testing for BD is not an emergency. There may be some urgency in circumstances where the person diagnosed as brain dead goes on to donate their organs, but one would assume that this is an unlikely scenario in cases where the family are contesting the diagnosis.

Persons suspected of being BD clearly lack capacity. In considering the matter of consent, a distinction must be drawn between persons aged under and over 16 years. For those under 16 years, the relevant question is whether parental consent to BD testing might be presumed to be required. We would suggest that there are reasonable grounds for such a presumption. In England and Wales, parental rights to consent to the medical treatment of their children are enshrined in s.3 of the Children Act 1989, while in Ireland, Article 42 of the Constitution strongly protects the rights of parents to make decisions on behalf of their children, only permitting this right to be supplanted in exceptional circumstances. The European Court of Human Rights (ECtHR) in *Glass v United Kingdom* appears supportive of this position, making it clear that where parents refuse consent, particularly in potentially end-of-life situations, then rights disputes of this kind should be determined by the courts and not by hospitals or doctors (at [83]).^20^ For those aged 16 and 17 years, the position is more complex. In England and Wales much of the Mental Capacity 2005 (MCA), including the statutory protection under s. 5, applies to those aged 16 years and over. This operates alongside common law duties of parental responsibility. The MCA Code of Practice (2007) states that where a young person (aged 16 and 17) lacks capacity to consent, healthcare staff may carry out ‘treatment or care’ with protection from liability (para. 12.17).^21^ The Code is, however, silent in respect of diagnosis. This leaves it open whether a requirement for parental consent continues to apply for persons in this category or whether the position under the MCA applies.

For adults lacking capacity, under s. 5 of the MCA acts in connection with the care or treatment of a person are statutorily protected provided that these are made on the basis of the best interests of the person (P). The issue of diagnosis is not explicitly addressed. However, operating on the basis that s. 5 protection extends to diagnosis, the determination of best interests requires that P’s past and present wishes and feelings, and the beliefs and values that would be likely to influence P’s decision if P had capacity, must be taken into account (MCA s4(6)). The MCA (s4(7)) requires consultation with a range of people to ascertain these, with the Supreme Court in *Aintree University Hospitals NHS Foundation Trust v James*,^22^ and the Court of Appeal in *R (Tracey) v Cambridge University Hospitals NHS Trust and Ors* 23 reinforcing this requirement in an end-of-life context. In the recent case of *St George’s v Casey* McDonald J further clarified the situation, stating that “[W]here there is a dispute about whether brain stem testing should be performed in respect of an adult who it is suspected has died, an application should be made to the Court of Protection”^24^.

In Ireland, the Assisted Decision Making (Capacity) Act 2015 (ADMCA) requires that account must be taken of the past and present will and preferences and of the values and beliefs of the relevant person, and that any intervention must be carried out in good faith and for the benefit of the person. As with the MCA, consultation with those close to the person is generally required. Both the MCA and the ADMCA also provide for advance refusals of ‘treatment’ (MCA ss.24–26; ADMCA, Pt.8). While the MCA does not define ‘treatment’, the ADMCA is clear that treatment includes any diagnostic intervention (ADMCA, s.82).

On the basis of this, we would suggest that the *prima facie* standard that applies is that consent from a parent or legal guardian is required for BD testing in the case of a child (clearly up to the age of 16, and possibly up to the age of 18 years); that an adult can refuse BD testing in advance; and that, in the absence of an advance refusal, the ascertainment of an adult’s wishes and feelings/will and preferences and beliefs and values is required before proceeding with BD testing. Where these standards cannot be met, testing should not proceed without referral to court.

### Case Law

The CJA article supports its argument with reference to statutory exceptions to the *prima facie* duty to obtain consent in various Canadian provinces ‘that probably apply to DNC’, and to case law in the USA, Canada and the UK. We have no comment to make on their interpretation of Canadian legislation save to say that there is no such legal exception in the UK or Ireland. We also have little to say on US case law except that elsewhere Pope has indicated that of the States where litigation on this matter has taken place (nine), there appears to be a consent requirement in three (Kansas, Montana and California).^25^ This is of little consequence to our argument except to highlight that there may not be jurisdictional uniformity in how courts think about this issue.

In respect of Canadian case law, the CJA article relies particularly on *Hayani vs McKitty*, which it states ‘is the only court ruling in Canada to directly address the DNC consent question’. It cites an order of the Superior Court of Ontario where Justice Shaw stated that: ‘Dr Baker shall perform the tests necessary to determine if Taquisha meets the neurologic criteria for death … including the apnea testing’ against the wishes of the family. In the order Justice Shaw explained that ‘per section 2 (1) of the Health Care Consent Act, this is an assessment and not treatment and consent of the SDM [surrogate decision-maker] is not required’. However, this latter detail is not included in the written final judgement. The CJA article argues that this deficit does not reduce its validity or relevance as precedent. However, as a general principle, we would suggest that a judicial decision not to include a matter in a final written judgment is entirely relevant to its precedential value.

There is a further problem. Justice Shaw issued the order on 28 September 2017. Taquisha had already been declared brain dead following testing on 20 September, with this finding confirmed by an independent expert on 22 September at the request of her family. Justice Shaw had also rejected the family’s claim that Taquisha’s Charter Rights in respect of her religious beliefs were infringed. Rather, she stated that as Taquisha was brain dead, she was not a legal person, and, thus, the Canadian Charter of Rights and Freedoms did not apply to her [at 207]. Given this, it is unsurprising that Justice Shaw provided the order that she did—peremptorily authorising a third set of tests on an individual who had already medically been declared dead and, thus, was not, in her view, subject to Charter rights. The Ontario Court of Appeal rejected Justice Shaw’s conclusion in respect of the applicability of Charter rights, finding instead that the appropriate methodological approach in a case where the outcome turned on the application of Charter rights was to presume that the applicant fitted within the category of Charter rights holders.^26^

While we cannot say that Justice Shaw’s order might have been different, if Taquisha had been presumed a legal person and rights bearer, or if the issue had arisen before a diagnosis of death had been made, we do point out that the CJA article makes quite expansive claims based on a perfunctory order likely influenced by the already delivered diagnosis of BD.

The CJA article also asserts that (r)ecent court decisions in the UK similarly confirm that consent is not required for DNC, referencing two of the Family Court’s judgments in *Battersbee*.^27^ We disagree that there is anything in *Battersbee* to support the conclusion that consent is not necessary. The background to the case is tragic if medically straightforward. Twelve-year-old Archie Battersbee suffered a prolonged period of hypoxic cardiac arrest, leading to a suspicion of BD. Archie’s parents refused to consent to the apnoea test, although they did agree to the other elements of BD testing, and for these to be assisted by any helpful ancillary test. The expert witness was of the opinion that, under the Code, the apnoea test had to be carried out, and that ancillary tests would not suffice ([2022] EWHC 1165 at [54]). As Archie’s parents had, therefore, effectively refused to consent, Barts NHS Trust made an application to the Family Court. Arbuthnot J declared it to be ‘lawful and in Archie’s best interests for brain stem testing to take place’, despite parental opposition ([2022] EWHC 1165 at [96]). Arbuthnot J does not specifically comment on the issue of consent, but rather proceeded on the standard basis that court referral is necessary in the absence of parental consent. This approach is entirely consistent with ECtHR jurisprudence as stated earlier, and also with the judegments in *Re A (A child*)^28^ and *Re M (Declaration of Death of Child*),^29^ both of which emphasise the role of the courts in the resolution of disputes around BD.^4^

When *Battersbee* returned to the Family Court in respect of a different matter some weeks later, Arbuthnot J cited with approval from *Manchester University NHS Foundation Trust v Fixsler & Ors* that:

The paramount consideration is the best interests of the child. The role of the court when exercising its jurisdiction is to take over the parents' duty to give or withhold consent in the best interests of the child. It is the role and duty of the court to do so and to exercise its own independent and objective judgment.^30^

Thus, Arbuthnot J appears to tacitly reinforce the status quo—consent from parents is required for any intervention and if it is not forthcoming, the matter is to be resolved by the court.

### BD is different

The CJA article outlines a variety of reasons why BD testing is different. One is that it does not trigger the prima facie duty to obtain consent because it is essentially diagnostic. As we have seen, BD testing requires highly intrusive interventions. Its diagnostic purpose does not render it qualitatively different to other medical procedures in terms of its impact on the right to bodily integrity. The breach of bodily integrity required to perform clinical tests for BD would not be permissible in a competent adult without their consent. To argue that the same interventions can be performed without consent (or consultation) in those who lack capacity due to severe neurological injury seems to us to be highly problematic as it relegates the right to bodily integrity of the profoundly disabled. As stated earlier, the diagnosis of BD is not an emergency, and so there is time to seek valid consent or engage in appropriate consultation. In this, it differs from a cardiac arrest, which is a true emergency. Thus, there is no tension between there being different obligations towards consent for assessment during cardiac arrest and assessment in cases of profound neurological injury—such a difference is entirely in keeping with regulatory and legal standards.

The CJA article also suggests that answering the question of whether someone is alive or dead is of fundamental importance; that there is a need to determine this in order to identify whether ongoing treatment is necessary or not; and that this need is such that it outweighs any requirement to acquire consent. A secondary issue is that providing ongoing interventions to someone who is dead is a waste of resources that might be better used elsewhere. It is difficult to argue with the importance of diagnosis or appropriate use of resources, even though whether BD represents ‘true’ death is the very point of contention that leads families into dispute with doctors and hospitals. However, even if one completely agrees with the importance of diagnosis, it remains quite a step to contend that this should override the human rights of individual patients. We would suggest that this level of paternalism represents an overreach of clinician authority, and an unwarranted retreat to the historical past of unquestioning deference to medical power that seems contrary to the ethos of recent jurisprudence in the UK and Ireland.^31^

### Deference to medical standards

The WBDP has robustly argued that, in the case of BD, medical guidelines and standards of practice are, or should become, the legal standard. The medical guidelines currently provide the correct pathway for BD diagnosis were accepted in both *McKitty* and by the Court of Appeal in *Battersbee*.^32^ However, there is a substantial difference between medical guidance that outlines a diagnostic approach and the role and authority of the courts. In the UK and Ireland, medical guidelines are advisory rather than statutory, and their validity in any given circumstance is subject to scrutiny and oversight by the courts should a question arise. This is as it should be—medical guidelines may be used by legal authority to judge individual actions but remain subordinate to the law which seeks to protect the interests of individual citizens. This is possibly best articulated by the Ontario Court of Appeal in *McKitty*:

The criteria for determining whether death has occurred is not a technical question that is indefeasibly the province of the medical profession, to which the common law must defer. The … criteria for death have not been accepted by the common law because medical practice is determinative, but because they have been judged by the common law to provide a sound answer to the question of how to determine whether a person has died … it does not follow that should a different medical practice emerge … the common law would be obliged to accept this as well [at 28].

The CJA article further argues that providing ongoing interventions to those individuals who are possibly brain dead negatively impacts ‘the integrity of the medical profession’, and that ‘forcing clinicians to act contrary to professional standards causes moral distress’. We find the argument that medical practice represents some universal system of integrity or values unconvincing. Even clinicians working solely in intensive care practice are not bound in some moral unity. In fact, there are considerable differences in end-of-life practices across jurisdictions, healthcare systems and even between doctors within individual hospitals. Thus, Wilkinson and Truog suggest ‘how a patient’s death is managed, and even potentially whether or not they die, is influenced by which physician happens to be on call’.^33^ The notion that the values of the individual clinician are more important than those of the patient, or even than their clinical condition, would seem deeply problematic, and certainly not indicative of some established system of professional integrity.

Moral distress among healthcare staff is obviously a matter of great concern. However, we would suggest that ongoing interventions cannot be regarded as problematic until the diagnosis of BD can be made and thus, such interventions should be regarded as necessary up to this point. The fact that doctors are highly unlikely to test for BD unless they reasonably believe that the person is brain dead does not change the fact that until such a diagnosis is made the person must be presumed to be alive and to have both the rights and interests of the living. This changes once the diagnosis is made,^5^ but while the person is presumed to be alive, we contend that the upholding of a patient’s rights should be a matter of significant moral concern for all clinicians. The approach documented in the account of the care of Taquisha McKitty despite ongoing litigation has much to recommend it in this regard.^34^

## Conclusion

In this article, we have argued against the proposition, which appears to be gaining some traction, that BD testing does not require consent. We contend that in the UK and Ireland, where testing involves a child parental consent is necessary, and where it involves an adult consultation with family members is required as to the person’s past and present wishes and feelings/will and preferences and their relevant beliefs and values. Where there is a dispute about whether BD testing should be performed in respect of an adult who it is suspected has died, an application should be made to the Court of Protection. Our argument here is not that BD testing should never take place in the face of opposition, but that in the event of a dispute the appropriate place to adjudicate such matters is the courts.

## Data Availability

Data sharing not applicable as no datasets generated and/or analysed for this study.

## Footnotes

The Guideline was developed in partnership by the Canadian Critical Care Society, Canadian Blood Services, and the Canadian Medical Association, and was funded by Health Canada.

We do not dispute (for the purposes of this article at least) the legitimacy of judicial acceptance of brain stem death as medically defined; for an argument for parliamentary intervention in this regard, see K. Choong, The Medio-Legal Development of Neurological Death in the UK. Singapore: Springer, 2022.

We refer to the UK in this article because the AoMRC is a UK-wide body, and the Code of Practice has UK-wide reach. However, we recognise that much of the analysis below refers to the jurisprudence of England and Wales.

The issue of parental consent had been raised in the Family Court in the first application in respect of *Re M*. Here the parents of a baby who had been diagnosed as brain dead argued that testing required their consent and that evidence from such a test was invalid in the absence of their consent. This argument was rejected by Lieven J who held [at 45] that the parents had known that BD testing was going to take place and had not objected. She also held [at 45] that there was no need for written consent to testing or for information in respect of the test to be written down. She did not however conclude on the more general issue of whether parental consent to testing was required. While this case was subsequently appealed, the matter of consent did not feature in the appeal.

The orthodox legal approach is that the dead have neither rights nor interests: see Re M (Declaration of Death) [2020] EWCA Civ 164, [49].
